# Do meat anti-consumption opinions influence consumers' wellbeing?–The moderating role of religiosity

**DOI:** 10.3389/fpsyg.2022.957970

**Published:** 2022-10-13

**Authors:** Ling Xie, Muhammad Faisal Shahzad, Abdul Waheed, Qurat ul Ain, Zunair Saleem, Mehwish Asghar Ali

**Affiliations:** ^1^School of Medical Information Engineering, Zunyi Medical University, Zunyi, China; ^2^Department of Marketing, Dr. Hasan Murad School of Management, University of Management and Technology, Lahore, Pakistan; ^3^Department of Finance, School of Economics, Zhejiang University, Hangzhou, China; ^4^International Business School, Shanxi Normal University, Xi'an, China; ^5^Putra Business School, University Putra Malaysia, Seri Kembangan, Malaysia

**Keywords:** consumer social responsibility, meat, anti-consumption, consumer health wellbeing, religiosity

## Abstract

The study aims to determine the role of personal factors, consumer social responsibility, and social marketing among meat anti-consumers. The study tests a model of anti-consumption using a sample of 597 (*n* = 597) participants from a cluster of young consumers through the distribution of the questionnaires in the Pakistani market. SEM employing the AMOS model for path relationships along with the Johnson-Neyman technique for moderation was mainly used. Results prescribe religiosity as the moderating driver of the anti-consumption of meat among young consumers in Pakistan. Consumer social responsibility is a robust antecedent, while social marketing is significantly documented for sustainability motives. Consumers apprise the personal health and environmental domain as an auspicious component for meat anti-consumption. The study reveals social marketing motivations for anti-consumption that eventually steers marketers and policymakers in shaping the concerned strategies. Our study delivers new insights into food anti-consumption behavior that provides guidelines for policymakers who heed consumer eating behaviors. The study is among pioneer work that establishes the moderating role of religious motivations and meat anti-consumption behavior among Muslim consumers to acquire healthy wellbeing.

## Introduction

The transformed paradigm in consumer consumption stresses the consumers to rethink their responsibilities toward society and the environment. Consumers' anti-consumption lifestyle for wellbeing is a promising concept in consumer literature (Malek et al., 2019). Studies are highly focused on the positive side of anti-consumption behavior (Arslan et al., 2018). Understanding the consumer's restrained consumption motives can boost health and human wellbeing (Chen et al., 2020). Health orientations and meat avoidance have been primarily documented as prevailing among young consumers (Nam et al., 2010; Gracia and Maza, 2015; Bogueva et al., 2017; Tosun and Yanar Gürce, 2018). Anti-consumption research has emerged mainly by focusing on anti-consumption behaviors over the past decades (Farah and Shahzad, 2020b).

Anti-consumption is “a function of a preference to consume one objeczsw2t over another.” In contrast, more profound types of anti-consumption attitudes involve “a resistance to, distaste of, or even resentment or rejection of consumption more generally” (Zavestoski, 2002). The study of anti-consumption behavior provides unique insights to marketing practitioners, policymakers, and researchers (Khan et al., 2019). Anti-consumption of meat holds a particular position in this domain because studies have reported that excessive meat consumption negatively affects consumer health (Bogueva et al., 2017). Similarly, animal welfare and sustainability concerns are also linked with meat consumption (Verbeke et al., 2010). There are several diets that could be harmful to the health such as vegan or vegetation diets sometime may lead to certain health issues along with several benefits for the human body (Ferraro et al., 2020; Soeters, 2020). There are also several other factors that may influence humans' health as the study the experts reported another factor of an anxiety disorder (Xiong et al., 2022). Despite this fact, meat consumption has increased recently (Food and Agriculture Orgnization, 2021).

To achieve consumer wellbeing, the research in terms of food marketing has increased the emphasis on restrained or anti-consumption behavior (Kashif, 2019). Consumers adopt such behaviors because of health concerns (Jin et al., 2017). Studies have reported that consumer personal factors and consumption practices always reveal new insights because consumer behavior is dynamic (Kaynak and Ekşi, 2014). Consequently, there is less evidence about consumer social responsibility and the role sensitivity to social marketing plays in the anti-consumption of meat. We have provided some essential factors drawing on the mechanism of meat anti-consumption. We also offered the moderating effect of religious connotation which effect muslins consumers. Study on anti-consumption has largely discussed the factors such as findings of Tosun and Yanar Gürce (2018) reported that consumers avoid meat due to health, price, and lifestyle factors.

Herman and Mack (1975) proposed the restrained theory (RT) and reported that individuals' avoidance is a conscious attempt enforced by environmental and health concerns (Johns et al., 2010). Other forms of anti-consumption include complaining behavior and various forms of brand avoidance (Shahzad et al., 2018). Personal factors can trigger anti-consumption. Meat holds a particular position in our life as it is an essential element of nutrition and traditional food that provides a lot of vitamins and energy. In contrast, WHO (Baron) report indicated that livestock officials in Pakistan and detected a virus among chickens and mounted over the illness's rapid spread. Although meat consumption has increased in the recent past, controversies prevail in the avoidance and consumption of meat in general because the meat is a significant component of the traditional diet and an enriched source of proteins (Adapa, 2018; Taufique and Vaithianathan, 2018). Moreover, young consumers do not have conscious consumption habits (Magnusson et al., 2003). Disagreement between increased consumption and health problems can create internal struggle and cognitive dissonance. The sense of health anxieties derived from continued eating is quickly replaced by restrained meat consumption. These opposing emotional states can also increase health perception among younger consumers (Arslan et al., 2018).

Consumers' health restricts the consumer likelihood of consuming unhealthy food (Zainuddin et al., 2013). Recent research on meat avoiders identified essential indicators of animal welfare, consumer health concerns, and sustainability (Sonoda et al., 2018). Health practitioners indicate the increasing number of patients suffering from chronic diseases resulting from unhealthy food consumption (Magnusson et al., 2003), endorsing global attention (Zainuddin et al., 2013). Social marketing efforts can trigger consumer attention (Aronowitz et al., 2018). Research on anti-consumption is inconsistent, and few studies identify anti-consumption antecedents (Allen et al., 2018; Arslan et al., 2018; Sudbury-Riley and Kohlbacher, 2018; Tosun and Yanar Gürce, 2018). Nonetheless, the present research will uncover the role of religious motivations attached to anti-consumption.

In order to achieve consumer welfare, understanding anti-consumption drivers are much needed (Allen et al., 2018; Arslan et al., 2018; Sudbury-Riley and Kohlbacher, 2018). Besides, these unhealthy and flagging lifestyles have further encouraged the call for anti-consumption research (Ozanne and Ballantine, 2010). Moreover, consumer social responsibility considers the increasing level of diseases and health problems that require attention (De Devitiis et al., 2012; Anderson, 2018b; Arli and Tjiptono, 2018b). Similarly, and from a consumer welfare perspective, poor food choices are a central issue as it leads to adverse health outcomes and is associated with sustainable consumption (Lim, 2017; Dermody et al., 2018; Wang et al., 2019). Lack of research is evident in this domain and highlights a knowledge gap in anti-consumption research (Bogueva et al., 2017; Sudbury-Riley and Kohlbacher, 2018). This research will consider anti-consumption behavior toward meat (Bonne et al., 2007; Bogueva et al., 2017; Sonoda et al., 2018; Tosun and Yanar Gürce, 2018). Moreover, no attempts have been made to examine the relationship between food anti-consumption and consumer social responsibility (Sudbury-Riley and Kohlbacher, 2018). The underlying research will undertake the present gap in the literature related to other research on meat avoidance behavior (Bogueva et al., 2017). A study by Farah and Mehdi (2021) examined consumers' insights and suggested more future work on consumers' perspectives is needed to unfold the influence in different perspectives and domains worldwide.

Because of the immense significance and higher recommendation of the worldly scholars, academicians, and practitioners on meat consumption and how it affects human health is a key inquiry to unveil globally (Tosun and Yanar Gürce, 2018). This study proceeds with such a theme with some additional factors such as religiosity and anti-consumption that got far less consideration from the researchers. Furthermore, this kind of work got little attention in developing nations particularly Pakistan which has great importance in the Asia region having the 6th highest populated position (Sonoda et al., 2018). Heath is a critical factor to emphasize for all the nations, therefore, it required higher attention to work on it to understand the insights within developing nations such as Pakistan. There are other motivations to study this domain are reported as follows. Unusual meat consumption can be associated with heart disease and digestive problems (Tosun and Yanar Gürce, 2018). Because animal welfare, environmental issues, and, more specifically, the health issues related to meat consumption act as physiological stimuli that can affect human welfare (Malek et al., 2019). Avoiding the usage of meat may not work because consumers often love to taste it. However, restrained consumption of meat can help achieve consumer and animal welfare (Tosun and Yanar Gürce, 2018).

## Literature review

### Personal factors and meat anti-consumption

Consumer personal factors that promote anti-consumption include internal factors such as an “individual's dispositions and his/her interpersonal attitudes that explain individuals' behaviors, thoughts, and emotions.” It includes individual characteristics (beliefs and traits) that are important factors affecting consumption practices. Empirically, evidence indicates that consumer hedonic characteristics such as personal beliefs and lifestyle may trigger healthy consumption (Contini et al., 2018; Farah and Shahzad, 2020b). The study of anti-consumption is always challenging and distinctive because it provides unique insights into consumers' attitudes and behavior that are not generally studied in conventional consumption studies (Ozanne and Ballantine, 2010; Buleandra et al., 2018). It can be observed from the past studies that consumers can undertake a variety of reasons to avoid certain products, which include environmental sustainability (Tosun and Yanar Gürce, 2018), religious sentiments (Al-Hyari et al., 2012a,b), and personal health concerns (Zainuddin et al., 2013).

There are several experts who worked to ensure the nexus between personal factors and the anti-consumption behavior of the consumers (Harnack et al., 1997). Meat products hold a particular position in our daily life as it is a vital element of nutrition and traditional food that provides a lot of vitamins and energy (Enderwick, 2009). Meat is an important foodstuff that provides nutation; a shift has been recognized in meat consumption in middle-income countries. The research has shown an increase in vegetable consumption among consumers. The genetically modified mass production of animals and food harms food security and consumer health. Some consumers perceive meat products as unhealthy because it is fattening and includes many saturated fats (Knight and Gao, 2009). Alongside consumer health concerns, sustainability concerns have raised the impact of these food production methods on the environment (Bogueva et al., 2017). Consumers' dietary choices are positively related to food provisions where non-vegetarian meals resulted in greenhouse gas emissions and powerfully impacted the environment. It is obvious to believe that consumption practices are directly linked to consumer personal factors such as beliefs and traits. Certainly, consumption or avoidance of a product is dependent on consumer individual dynamics. Accordingly, the researchers postulate as follows.

***H***_**1**_*:* Personal factors (e.g., lifestyle and economic factors) positively affect consumer meat anti-consumption behavior.

### Consumer social responsibility and meat anti-consumption

Consumer social responsibility can be defined as “the conscious and deliberate choice to make certain consumption choices based on personal and moral beliefs (Arli and Tjiptono, 2018a,b).” It can also be described as “the application of instrumental, relational, and moral logic by an individual, group, corporate and institutional agents seeking to influence a broad range of consumer-oriented responsibilities”. The authors suggested that consumers have two specific responsibilities: consumer ethics and consumer social responsibility (Bogueva et al., 2017). Consumer social responsibilities “Include” not harming society and acting proactively for social benefits, including consuming and disposing of products and services (Baron, 2013; Nicola Sneddon et al., 2014). It may also include responsibility toward the environmental domain, stakeholder domain, and consumer domain at large (Bogueva et al., 2017). Food consumption is a significant element that builds up sustainability in the food supply. Poor food consumption has a substantial effect on society and individual wellbeing. Dietary patterns worldwide are changing (Hingley et al., 2013; Chen et al., 2019). There are some personal, social, and environmental factors that play an essential role in food intake and avoidance (Chen et al., 2019). As the links between health consciousness and dietary practices have emerged, consumer attitudes and personal beliefs become important in consumption decisions (Enderwick, 2009). There are several studies have reported consumer resistance to meat product consumption from different perspectives and themes of the world (Allen et al., 2018). Several reasons might count for meat product avoidance, such as lactose intolerance or casein allergy, cultural norms, religiosity, or fat they contain in general. Consumers' resistance and anti-consumption represent diverse literature where consumer experience and actions are foregrounded and explain resistance in behavior (Nicola Sneddon et al., 2014; Arli and Tjiptono, 2018a).

Most previous studies seek to establish consumer social responsibilities domains in fast-moving consumer goods (Arli and Tjiptono, 2018a). No attempts have been made to examine the relationship between food anti-consumption and consumer social responsibility (Sudbury-Riley and Kohlbacher, 2018). It is assumed that marketers need to understand these differences for effective marketing strategies in different world regions (Enderwick, 2009). Past research is evident that consumers may have different decision-making styles for each product category; most of the studies have investigated consumer decision-making with specific types, including everyday products, online shopping, and food products buying (Hartmann et al., 2016). This research considers the literature gap related to casual research on antecedents of food-related anti-consumption behavior. It fills it by exploring other variables (religiosity and consumer social responsibility). Thus, it will help the consumer make a careful decision (Allen et al., 2018). The conceptualization and development of Social Learning theory and health beliefs on consumers' cognitive and personality characteristics will add significant value (Chen and Kong, 2009; De Devitiis et al., 2012).

Animal welfare and anti-consumption have been documented where consumers have shown resistance to killing thousands of animals (Sonoda et al., 2018). The researchers argued that lack of concern toward animal welfare is a form of unethical action governed by individuals. It can also be seen from the previous studies that most of the meat anti-consumers take animal welfare as an essential antecedent of meat avoidance (Hingley et al., 2013; Sonoda et al., 2018). Animal welfare plays a significant role in anti-consumption behavior, combined with the different two motivations, i.e., health and environmental concerns, and the other two motivations, i.e., health and environmental concerns, animal welfare plays a significant role in anti-consumption behavior. Tosun and Yanar Gürce (2018) report consumer lifestyle and health concerns are more significant motivators. Research on animal welfare and anti-consumption is not that promising in countries such as turkey. “An in-depth study of other regions will explore new insights.” Accordingly, consumption practices and the role of consumer social responsibility drive the restrained consumption behavior that eventually leads to form the following assumption.

***H***_**2**_***:*** Consumer social responsibility will positively affect meat anti-consumption behavior.

### Role of social marketing

Social Marketing is a form of marketing that is rapidly growing and contribute to consumption reduction. At the same time, societal marketing undertakes commercial marketing in pursuit of social goals (Lefebvre, 2013). Therefore, social marketing is an approach to planned social change where consumer consumption practices are linked. Social Marketing is a marketing mix pyramid that is a dominant perspective in social behavior research (Felix et al., 2017; Lim, 2017). The primary goal of social marketing is to strive for public health, which is a social goal of improved consumers' welfare (Dibb and Carrigan, 2013; Enyinda et al., 2018). For example, to reduce the risk of Smoking, and drinking social marketers can aim at wellness services for consumer wellbeing (Heiman et al., 2019). Similarly, consumer meat anti-consumption is a phenomenon where the consumer considers weight management, environmental sustainability, and animal welfare (Allen et al., 2018; Shareef et al., 2019). Social Marketing can add value to consumer wellbeing by enhancing the role of anti-consumption behavior. Consumption of a product is far easier to advertise in media. It is challenging to make consumption reduction that is not appealing to consumers, and policymakers also find it to advertise anti-consumption campaigns for health and sustainability orientations. Future consumption reduction could address social marketers and policymakers in health risks and economic terms (Lim, 2017; Sanclemente-Téllez, 2017).

In terms of appealing to consumers' truth, social marketing campaigns are acceptable like anti-consumption of Smoking. Social marketing of green product consumption may lead to consumer welfare in the long run. Social Marketers can encourage anti-consumption with the help of emotional and symbolic meanings of products. Although there are some disagreements about whether social marketing can enhance anti-consumption behavior and consumer social responsibility in consumer behavior research and practice can be appropriate depending on the objective of the study (Sen et al., 2006; Peattie and Peattie, 2009; Felix et al., 2017). Moreover, the study on the value of health and wellbeing has given importance to consumer education and management in value creation (Shahzad et al., 2015; Heiman et al., 2019). Despite this, limited research is evident on consumer anti-consumption and social marketing, as highlighted earlier, indicating a significant gap in colonial marketing literature since this will be helpful in achieving improved wellbeing of consumers (Dibb and Carrigan, 2013; Lim, 2017; Aronowitz et al., 2018).

It is reported by experts that anti-consumption and food-related welfare would potentially have wide applications among food marketers, policymakers, and consumers (Ulph and Ulph, 2018). Traditional food choices positively affect consumer health, such as consuming functional foods, but the study of anti-consumption would make a difference in levels of welfare (van Riemsdijk et al., 2017). It would determine the antecedents of consumer welfare separated from traditional indicators (Chen and Kong, 2009; Kim, 2019). Marketers might use this information to access the segments of anti-consumption and guide the future allocation of marketing resources (Gram et al., 2015; Akaichi and Revoredo-Giha, 2016). This paper will attempt to investigate consumer welfare supported by limited empirical evidence and answer how consumer welfare can drive by the anti-consumption of food (Jayawardhana, 2013). Based on the above-précised discussion, we eventually proposed the following hypothesis.

***H***_**3**_***:*** Social marketing will positively affect meat anti-consumption behavior.

### Anti-consumption behaviors and health wellbeing

Based on the above-cited literature, we have found that personal factors, consumer social responsibility, and social marketing are essential drivers of meat anti-consumption, which can help to increase human health and wellbeing. However, highly health-conscious consumers are more likely to limit their unhealthy food consumption even if they enjoy such foods, especially meat consumption (Heinonen et al., 2013; Tosun and Yanar Gürce, 2018). Studies imply that excessive consumption of meat results in high blood cholesterol levels. These negative feelings interrupt consumer attitudes and create disappointment and disconnection with consumption (Heinonen et al., 2013). The latter, in turn, reduce consumption, which breeds anti-consumption tendencies and behaviors (Jayasimha and Srivastava, 2017). Studies have reported Generation Y as having more spending and being savvy consumers. This is a key marketing segment in the food industry due to their consumption habits and lifestyles (Farah and Shahzad, 2020a,b). They are also health conscious. As a result, the researchers stipulate the following hypothesis:

***H***_**4**_***:*** Meat anti-consumption behavior is positively related to consumer wellbeing.

### Moderating role of religiosity and meat anti-consumption

According to intrinsic religiosity, an individual is strongly committed to their religion. Social and reference groups may influence extrinsic religiosity to meet personal needs (Arli and Tjiptono, 2018b). A strong association has been found between intrinsic religiosity and health-related consumption and support for consumer welfare. Religion holds a strong position in the lives of believers and enforces doing things, limits lifestyles, and delivers what, why, and how items to be consumed (Minton et al., 2015). A study on Muslim consumers in terms of halal meat consumption found that personal attitude, social influence, and perceived control predict intentions toward halal meat consumption (Bonne et al., 2007; Cleveland et al., 2013). Evidence provided from previous studies that religion positively affects attitude and behavior (Sonoda et al., 2018), and it predicts food choices in many cultures. The viewpoint of religion in consumption has been discussed in detail through its role in consumers' anti-consumption choices is unclear. Anti-consumption is linked with religion (for example, muslin consumers do not consume pork meat because of strict religious considerations) (Cleveland et al., 2013).

Researchers have studied the effect of religious animosities on purchase intention, and findings revealed that ecclesiastical endorsement is very appealing to a particular focus group. Still, in the same way, it leads to a decrease in purchasing intent among other segments (Souiden and Rani, 2015). Studies have argued that religious-based endorsement reduces the acceptability of products from majority segment groups. Authors concluded that religion effect in several ways of consumer lifestyles affects their choice of behavior (Asraf Mohd-Any et al., 2014). Food choice motives of different ethnic groups in Malaysia revealed no difference in food choice except familiarity. In halal consumption, Muslim consumer food choices affect their decisions (Henderson, 2010; Asraf Mohd-Any et al., 2014).

Furthermore, studies have indicated that religiosity affects values (Katz-Gerro and Jaeger, 2012), and values influence consumers' attitudes and behavior (Almossawi, 2014). It can be derived from the above discussion that consumer attitudes toward anti-consumption can be linked to consumers' religious beliefs (Engelund et al., 2007). Consumers with high intrinsic religiosity tend to support social initiatives (Arli and Tjiptono, 2018b). Heiman et al. (2019) state adherence to the religious dietary pattern in another region of the world would add value to the anti-consumption studies. This study will explore the effect of food taboos on consumption patterns. In this concern, social dilemma theory would explain the underlying mechanism. Therefore, we hypothesize the following hypothesis.

***H***_**5**_***:*** Religiosity will moderate the relationship between personal consumer factors and anti-consumption behavior.

### Theoretical model

**Figure 1:**
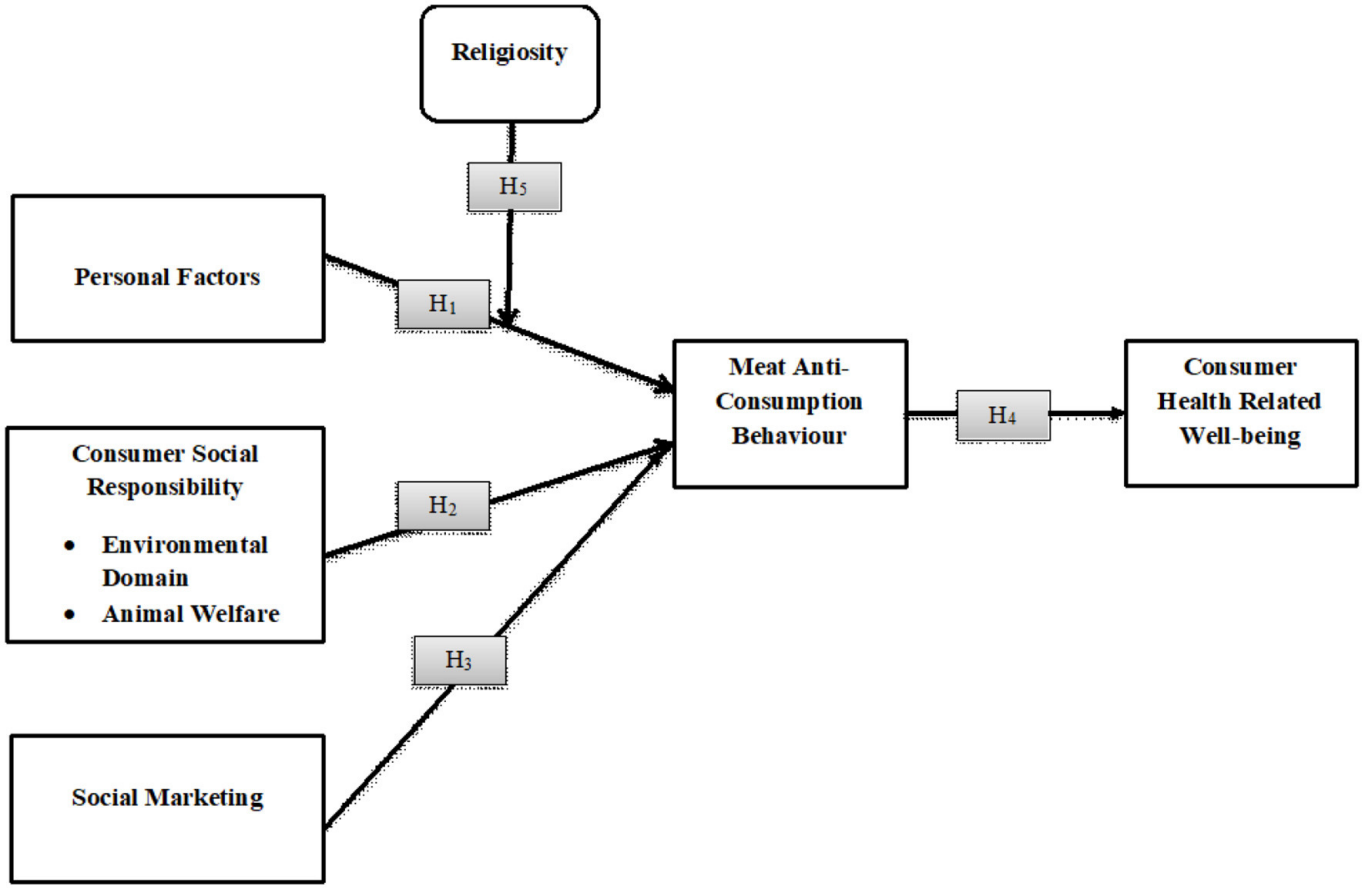
Theoretical model.

This model (Figure 1) is based on the restraint theory proposed by Herman and Mack (1975) and (Ogden, 1994). The model indicates that “controlled eating is a conscious practice enforced by individuals' personal preferences as well as their environment” (Farah and Shahzad, 2020a,b). This theory was developed to restrict consumers' food intake for weight control. It evaluates both causes and consequences, which help limit excessive consumption for obesity prevention.

## Methodology

This study aims to provide consumer motivations toward meat anti-consumption by considering religiosity and consumer social responsibility concerns to achieve consumer health-related welfare. Anti-consumption is not repeated consequently limited studies are evident in meat anti-consumption behavior.

### Data collection and measures

A structured questionnaire was used to collect data (Shahzad et al., 2015; Islam et al., 2019). This mode of data was collected using two methods such as physical method and the online method. First, in the physical method, we personally met the participants during different time periods and requested them to fill out the questionnaire. Second, in the online method, we shared the link to the questionnaire using emails of the different participants and requested them to fill out the online questionnaire. It took several months to obtain the data by both means of data collection. The questionnaire consisted of two sections. The first section included demographic questions. In the proceeding section, respondents were asked to rate the importance of various factors in meat anti-consumption behavior through academically validated scales adopted from vast literature. Data were collected using a convenience sampling technique (Farah and Shahzad, 2020b).

The target population of the study was the “Generation Y” cohort. It is reported in the literature that the vast majority of Gen Y claim to eat or intend to eat healthy food. Though, their consumption styles would suggest otherwise (Shahzad et al., 2022). This cohort is characterized by health consciousness compared to older cohorts. They are highly involved and concerned about their individual and social welfare purchase decisions (Anderson, 2018a). According to sociological literature, it comprised social responsibility and shared values in its youth. Consumers' data was gathered from a structured questionnaire and then completed using a survey throughout the country (Sudbury-Riley and Kohlbacher, 2018). The consumer tracking was taken place by personal administration of the questionnaire (Tosun and Yanar Gürce, 2018). Seven hundred thirty-four responses were collected, and, after screening, *n* = 597 resulted in the final sample. Multiple items were drawn from existing literature for each construct. Articles were assessed on a 7-point Likert scale, indicating one strongly disagree to 7 strongly agree. There are several experts who used similar scales in their studies to recording the consumers' responses; therefore, our study is consistent with such studies (Waheed et al., 2020). To measure the key outcome variables (Anti-consumption behavior, consumer welfare), meat anti-consumption studies reflected health, environmental sustainability, lifestyle, and economic concerns to remember the context of this study (Tosun and Yanar Gürce, 2018). To measure consumer health wellbeing (Henson and Traill, 2000), nine items were used based on food-related consumer welfare. Items from Arli and Tjiptono (2018b) were used to measure consumer social responsibility. To calculate religiosity, items from Arli and Tjiptono (2018b) were used. To measure the moderating effect of social marketing, five items from Arli and Tjiptono (2018a) were used. Using AMOS, SEM was applied to test the hypothesis. To ensure the validity and reliability of the constructs, confirmatory factor analysis (CFA) was used to test the measurement model before testing the structural model, followed by Hair et al. (1998) two-step approach. To deal with the missing values, Maximum Likelihood estimation was used. Items loading values < 0.6 were dropped.

## Results

The results of CFA indicating an overall acceptable fit in Table 1 (χ 2 = 265.62, df = 151; RMSEA = 0.039; CFI = 0.933). All measurement items significantly loaded their estimated latent construct *p* = 0.001. All constructs' composite reliability also meets the threshold reliability requirements (0.80 or above) (Bagozzi and Yi, 1988). The average variance extracted (Zavestoski) for all latent constructs met the cut-off value of 0.50 (Fornell and Larcker, 1981), ensuring the discriminant validity for all constructs in the model. Discriminant validity was tested using (Fornell and Larcker, 1981). According to Sekaran and Bougie (2011), discriminant validity occurs when “two variables are predicted to be uncorrelated, and the scores obtained by measuring them are empirical.” Results obtained in Table 3 fulfilled the required validity and reliability criteria. The present study's constructs and measurement model items were appropriate for testing propositions. CFA (confirmatory factor analysis) and AMOS were used to test the relationships (Ramadan et al., 2019).

**Table 1:** Measurement model.

**Measure**	**Standardized**	**Standard**
	**factor loading**	**errors**
**Food anti-consumption (AVE; CR) (1 strongly disagree; 7 strongly agree) I do not eat meat, because…**	(0.62, 0.91)	
FAC1. It is expensive	0.85	0.02
FAC2. My income does not allow me to purchase meat	0.87	0.01
FAC3 I do not eat meat usually	0.85	0.01
FAC4 I cannot find meat while shopping	0.77	0.01
FAC5. Its taste is not good	0.89	0.02
FAC6I cannot find it in restaurants	0.77	0.01
FAC7it is not easy to cook meat	0.85	0.02
FAC8. My friends/family do not eat it	0.77	0.01
FAC9. Being slim and fit, maintaining bodyweight	0.87	0.01
FAC10. Concerns about artificial growth of animals, hormones, and drugs used in animal production, lacking free-range animals	0.82	0.01
FAC11. Controlling the quantity of meat intake and replacing it with fruit and vegetables to avoid diseases associated with meat consumption	0.73	0.01
FAC12. It is hard to find high-quality meat (hormones used in animal production, animals kept in		
cages, hygiene concerns)	0.88	0.02
**Consumer social responsibility (AVE; CR) (1 strongly disagree; 7 strongly agree)**	(0.82, 0.91)	
CSR1. Safety of food is good these days	0.89	0.01
CSR2.In general, I am satisfied with the convenience of food available today	0.77	0.02
CSR3. Today's food contain nutritional value	0.85	0.01
CSR4.Price of today's food is acceptable	0.87	0.01
CSR5. I like taste of food available these days	0.85	0.02
CSR6, In general, I am satisfied with the ethics of the way in which food is produced today	0.85	0.01
CSR7. In general, I am satisfied with the Choice of foods available today	0.85	0.01
CSR8, In general, I am satisfied with the behavior of food companies today	0.77	0.01
CSR9. In general, I am satisfied with where most of the food available today comes from	0.87	0.02
CSR10. Avoiding meat because of animal welfare	0.82	0.01
CSR11. Not eating meat because it is better for the environment	0.77	0.02
**Religiosity (AVE; CR) (1 strongly disagree; 7 strongly agree)**	(0.79, 0.89)	
R1I usually read about my religion	0.87	0.01
R2. It doesn't matter much what I believe so long as I am good I.	0.73	0.01
R3. It is important to me to spend time in private thought and prayer.	0.78	0.01
R4. I have often had a strong sense of God's presence.	0.91	0.01
R5. I try hard to live all my life according to my religious beliefs.	0.87	0.01
R6. Although I am religious, I don't let it affect my daily life I.	0.87	0.01
R7. Although I believe in my religion, many other things are more important in life I	0.77	0.01
R8. I go to a religious service because it helps me to make friends.	0.87	0.01
R9. I go to a religious service to spend time with my friends.	0.85	0.01
R10. I go to a religious service because I enjoy seeing people I know there.	0.85	0.01
R11. What religion offers me most is comfort in times of trouble and sorrow.	0.81	0.01
R12. I pray mainly to gain relief and protection.	0.87	0.02
R13. Prayer is for peace and happiness.	0.88	0.01
**Consumer wellbeing (AVE; CR) (1 strongly disagree; 7 strongly agree) I do not eat meat, because…**	(0.81, 0.92)	
CW1. I want to reduce energy consumption	0.87	0.01
CW2. I want to reduce emissions like CO2	0.87	0.02
CW3. I want to prevent waste	0.77	0.02
CW4. I want to recycle	0.87	0.01
CW5. I want to dispose of waste correctly	0.85	0.02
CW6. I want to invest in research and development regarding environmental protection	0.77	0.01
CW7. I want Corporate environmental protection standards are higher than legal requirements	0.87	0.02
**Social marketing (AVE; CR) (1 strongly disagree; 7 strongly agree) I do not eat meat, because…**	(0.79, 0.90)	
SM1. I would be willing to pay a little more to buy a product from a company that has a good record on hiring and promoting environmental sustainability	0.85	0.01
SM2. I would be willing to pay a little more to buy a product from a company that has good environmental practices	0.87	0.02
SM3. I would be willing to pay a little more to buy a product from a company that has a good record of hiring and promoting ethnic minorities	0.87	0.02
SM4. I would be willing to pay a little more to buy a product from a company whose television advertising does not glamorize violence	0.86	0.01
SM5.I would be willing to pay a little more to buy a product from a company that does not use animal testing	0.85	0.02

Table 2 shows correlations between AVE scores and square roots (Hair et al., 2016). The composite reliability (Lefebvre) for each scale ranged from 0.80 to 0.95–all above the recommended threshold suggested in the extant literature (Hair et al., 2016).

**Table 2:** Discriminant validity results.

**Factor**	**1**	**2**	**3**	**4**	**5**
Consumer social responsibility	1				
	(0.84)				
Religiosity	0.61	1			
		(0.83)			
Meat anti-consumption	0.47	0.45	1		
			(0.87)		
Consumer health wellbeing	0.40	0.44	0.33	1	
				(0.82)	
Sensitivity to social marketing	0.55	0.62	0.43	0.44	1

Results indicated in Table 3 for testing structural model resulted in an acceptable fit (χ2 = 26 8. 01, d f =151; RMSEA = 0.040; CFI = 0. 954). H1 and H2 are supported: consumer personal factors positively relate to anti-consumption consumption (0.289, t = 4.0 54) and consumer social responsibility also positively relate to anti-consumption (0.216, t = 3.341) supporting H2. Similarly, sensitivity to social marketing positively relates to Meat Anti-consumption (0.264, t = 4.170). Finally, consumer meat anti-consumption was positively related to consumer health wellbeing (0.181, t = 5.113). Table 3 summarizes the findings in relation to the hypotheses. Regarding the control variables such as age has a slightly significant positive effect on food anti-consumption (0.071, CR = 1.470). Overall, the model explains 29.1% of the variance of food anti-consumption. Gender does not have a *significant* effect on meat anti-consumption.

**Table 3:** SEM estimates.

	**Path**			
**From**	**To**	***p-*** **Value**	**Hypotheses**	**Standardized estimate t**
				**(CR)**
Personal factors	Meat anti-consumption	0.01	H1	0.289 (4.054)
Consumer social responsibility	Meat anti-consumption		H2	0.216 (3.341)
Social marketing	Meat anti-consumption	0.01	H3	0.264 (4.170)
Meat anti-consumption	Consumer health related wellbeing	0.02 0.01	H4	0.181 (5.113)
CR = 1.96 (α = 0.05 level)				

### Moderation analysis

**Figure 2:**
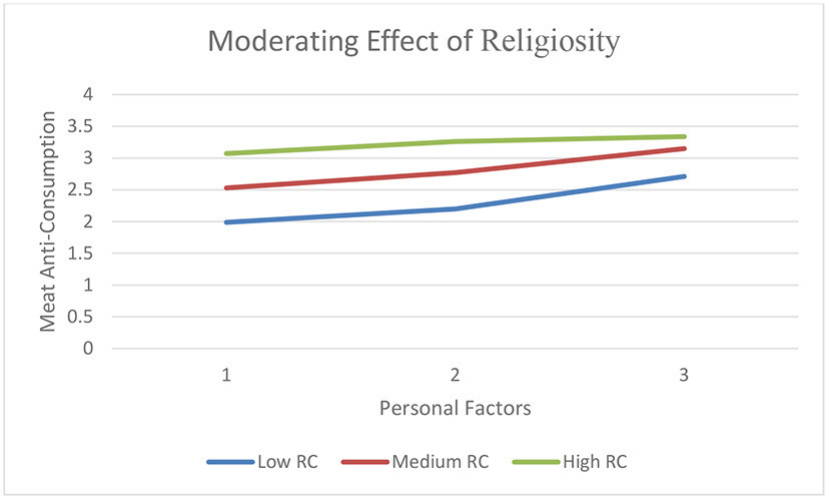
A Plot of PF, Personal Factors; MAC, Meat Anti-consumption, vs. RC, Religious Connotations the Moderator with Meat.

Hayes (2017). Process macro, Model 1, in SPSS 21.0 was used for data analysis. To test H5 among young consumers, the regression model produced the following statistics: R^2^ = 0.13; F (2.212) = 4.26, *p* = 0.001. Results revealed significant have shown values of the moderated indirect and direct effects of RC in influencing meat anti-consumption (H5) are shown in Table 4. Low and medium levels of RC have a significant positive indirect effect (indirect effect (low) = 0.79, CI.95 = 0.17, 1.21; and indirect effect (medium) = 0.67, CI.95 = 0.016, 0.77). However, for higher values of RC behavior, there is an insignificant relation (indirect effect [high] = 0.28, CI.95 = −0.17, 0.61) for H5. Results found the conditional indirect effect is positive but declines as RC increases. Figure 2 shows the indirect and direct impact (personal factors and meat anti-consumption) at varying levels of RC with a 95% confidence interval. Figure 2 suggests that the indirect effect between personal factors and meat anti-consumption is conditional upon religiosity among Pakistani consumers. At a certain level of RC, meat anti-consumption motives also decrease. The Johnson–Neyman technique results are shown in Table 5. Results suggest that the relationship between personal factors and meat anti-consumption is significant only up to a certain level (i.e., 0.92), beyond which this relationship becomes insignificant.

**Table 4:** Conditional effect of (IF) on (MAC) at different value levels of the moderator (RC).

**Conditional direct effects**	**W**	**Effect**	**se**	**t**	**p**	**LLCI**	**ULCI**
Low RC	3.32	0.79	0.24	3.14	0.01	0.17	1.21
Average RC	3.14	0.67	0.21	2.54	0.01	0.016	0.77
High RC	3.26	0.28	0.19	4.412	0.60	−0.17	0.61

PF, Personal Factors; MAC, Meat Anti-consumption; R, religiosity.

**Table 5:** Conditional effect for different values of the moderator (RC) using the Johnson-Neyman technique.

**RC**	**Effect**	**se**	**t**	**p**	**LLCI**	**ULCI**
4.88	0.96	0.43	2.22	0.01	0.11	1.89
5.23	0.90	0.40	2.43	0.01	0.13	1.77
4.93	0.89	0.36	2.45	0.02	0.15	1.67
4.62	0.83	0.33	2.82	0.01	0.17	1.56
4.31	0.81	0.32	2.72	0.03	0.20	1.44
3.99	0.74	0.28	2.88	0.02	0.2	1.35
3.68	0.70	0.23	3.13	0.01	0.22	1.26
3.37	0.63	0.21	3.17	0.01	0.21	1.18
3.06	0.62	0.16	2.96	0.02	0.2	1.08
2.74	0.58	0.14	2.87	0.03	0.18	1.12
2.43	0.54	0.15	2.65	0.02	0.17	0.97
2.29	0.46	0.15	2.35	0.01	0.11	0.93
2.08	0.44	0.15	2.98	0.02	0.06	0.89
1.79	0.42	0.15	1.54	0.05	0.08	0.87
1.49	0.40	0.17	1.52	0.02	−0.01	0.86
1.18	0.37	0.18	1.31	0.05	−0.02	0.85
0.87	0.36	0.21	0.92	0.11	−0.17	0.77
0.56	0.29	0.23	0.71	0.24	−0.23	0.76
0.25	0.23	0.25	0.53	0.30	−0.34	0.82
−0.06	0.19	0.28	0.42	0.39	−0.44	0.85
−0.37	0.17	0.31	2.52	0.45	−0.51	0.86
0.46	0.11	0.34	2.43	0.54	−0.59	0.87

To investigate the interaction of PF, Personal Factors; MAC, Meat Anti-consumption and R= religiosity on MAC= Meat Anti-, the PROCESS MACRO incorporating the Johnson-Neyman technique was utilized, using arbitrary points of the moderator (i.e., RC). The results reveal all ranges of the moderator in which the focal predictor (PF) is a significant predictor of the outcome (i.e., MAC). Highlighted values in italics indicate that the conditional effect was a significant predictor of MAC.

## Discussion and implications

The religious world may support overcoming the consumption ideology where different factors are associated with consumption such as social economic or lifestyle (Rayner and Easthope, 2001). This study has examined the phenomena of meat anti-consumption to achieve consumer health and wellbeing. The evidence was collected from Pakistan to explore in Table 6 the role of religious motivations. In today's fast-paced society, consumers face health problems due to inconsistent and binge eating habits. This is due to the modern consumer's lifestyle and related societal changes. However, this study indicates that personal and social problems related to meat consumption may breed undesirable feelings that arouse anti-consumption tendencies. The social marketing and consumer social responsibility impact on anti-consumption are 2-fold. First, social marketing and food consumption have been discussed in detail. For example, consumers avoid meat for social wellbeing motives. This study validates the density of these phenomena, suggesting personal consumer motivations and social marketing social pressure should be included in future research to find novel findings in different contexts. Understanding meat anti-consumption will help managers better equip consumers in the future, and realistic policymaking can be achieved. People avoid meat for health concerns than other testified reasons. Consumer social responsibility positively impacts meat anti-consumption behavior was expected. However, these results also attain consumer welfare. Given the consumer social responsibility and anti-consumption gap, it is believable that environmental and social responsibility concerns are better indicators of anti-consumption. Despite the fact, that animal welfare has been reported less and refuses the previous findings (Tosun and Yanar Gürce, 2018). The positive relationship between consumer social responsibility and anti-consumption supports that older consumers who think their consumption behavior will contribute to social action (Baskentli et al., 2019).

**Table 6:** Hypothesis supported/ not supported.

**Hypothesis**	**Statements**	**Results**
H_1_:	Personal factors effect positively related to their consumer meat anti- consumption behavior.	Supported
*H_2_*	Consumer social responsibility will have positive effect on meat anti-consumption behavior.	Supported
H_3_:	Social marketing will positively affect meat anti-consumption behavior.	Supported
H_4_:	Meat anti-consumption behavior is positively related to consumer health-related wellbeing	Supported

This study has managerial implications for the management to understand the insights about the anti-consumption behavior of the consumers before fulfilling their market needs. Since we know that the religious impact on meat anti-consumption was higher among personal motives among Pakistani consumers. Past research has undergone personal and health-related issues of anti-consumption, indicating a gap for further investigation (Galvagno, 2011). The integration of social marketing into the model has shaped some novel findings. Instead, consumer social responsibility directly impacts the anti-consumption of meat; results show that social marketing efforts toward the environment and animal welfare encourage anti-consumption behavior. These results make perfect sense where such marketers who vigorously promote this belief that human activities are severely abusing the environment and the background essence of social responsibility toward the anti-consumption of such products can increase. Similarly, such consumer behavior leads to consumer welfare (Ulph and Ulph, 2018). A study by Sudbury-Riley and Kohlbacher (2018) has reported a positive relationship between age and anti-consumption of food products and suggests further investigation. Economic and lifestyle concerns were the least important in anti-consumption (Tosun and Yanar Gürce, 2018). Equally interesting is that social marketing moderates the relationship between consumer social responsibility and anti-consumption (Anderson, 2018a). The findings suggest that social marketing would be a viable source to enhance consumer welfare. This research has identified a cluster of older consumers who are likely to engage in anti-consumption, keeping in view the external social religiosity as an important motivator. Previous researchers established that meat anti-consumption results from consumers' lifestyles, animal welfare, and environmental sustainability (Tosun and Yanar Gürce, 2018). Our study has identified factors other than personal motives and found that consumer social responsibility can affect meat anti-consumption and that social marketing has a strong effect (Bogueva et al., 2017). Results have shown a positive impact of consumer social responsibility toward anti-consumption due to moral avoidance for humans and the environment. Interestingly, the results revealed a moderating effect of religiosity between meat anti-consumption and human welfare. We can say that meat-anti consumption for the purpose of consumer welfare can be enhanced through religiosity. The research has also made a contribution to the literature on social marketing and meat anti-consumption. An in-depth understanding of adult consumers' anti-consumption behavior would offer improved strategic planning to marketers and policymakers. The generation Y cohort would add value to the anti-consumption research in a future study. Moreover, additional social implications are reported.

In sum, we find that individual lifestyles that reflect an individual's behavior are in line with previous research. In addition, this study put forward that meat anti-consumption for welfare results from social responsibility and social marketing that can endorse such behaviors. Some recent studies have reported similar results (Shahzad et al., 2019; Farah and Shahzad, 2020a). The study has put forward an important implication for policymakers to plan better approaches to consumer welfare and offer a public policy to practitioners for the sake of promoting sustainable consumption through social marketing efforts (Aronowitz et al., 2018).

## Conclusion

Restraint consumption to meet is sometimes necessary for human and environmental sustainability. There are few studies that witnessed ethical purchasing and anti-consumption behavior. Anti-consumption has an influence on human and ecological sustainability. Policymakers have endeavored with a considerable determination to establish sustainable consumption. This empirical study attempts to accomplish consumer social reasonability and religious decisions that drive anti-consumption in a different manner. Social marketing contributes to enhancing the understanding of said phenomena to achieve consumer health-related wellbeing—a higher level of extrinsic social religiosity and consumer social responsibility results in anti-consumption. Interestingly environmental sustainability has a more significant impact on the anti-consumption of meat whereas animal welfare has not been that much encouraging. Finally, an essential social marketing role has emerged in the current era that suggests favorable social activities can enhance sustainable consumption. This study has focused on the young consumers of Pakistan, a distinct segment of society who powerfully persuasive effect on society. Some studies have suggested research into aging adults because of their social, economic, and political changes (Sudbury-Riley and Kohlbacher, 2018). Mainly, the research has increased the understanding of anti-consumption, explicitly contributing to religious motivations and critical social marketing efforts. The reason to study anti-consumption was to attain consumer welfare (van Riemsdijk et al., 2017) by focusing on young consumers (Sudbury-Riley and Kohlbacher, 2018).

## Limitation and future research

This study has limitations of gender difference and limited sample size. Future studies on other cohorts could provide exciting insights into anti-consumption motives among non-muslin consumers. The moderating effect of gender and education could have exciting findings in the future since these factors were massively used in distinct health-related studies. Forthcoming, studies could utilize longitudinal studies to further expand the concept. Our study requires a natural setting so the experimental study could provide new insights in the future. Other food categories can provide a better understanding and aid to validate the current findings on a larger scale.

## Data availability statement

The raw data supporting the conclusions of this article will be made available by the authors, without undue reservation.

## Ethics statement

Ethical review and approval was not required for the study on human participants in accordance with the local legislation and institutional requirements. Written informed consent for participation was not required for this study in accordance with the national legislation and the institutional requirements.

## Author contributions

Conceptualization: MS and XL. Investigation: AW. Modeling and analysis: QA, ZS, and MA. Writing—original draft: MS. Writing—review and editing: XL. All authors contributed to the article and approved the submitted version.

## Funding

This project was funded by the 2018 Special Project for Cultivation and Innovation of New Academic,Qian Platform Talent [2018]5772-012.

## Conflict of interest

The authors declare that the research was conducted in the absence of any commercial or financial relationships that could be construed as a potential conflict of interest.

## Publisher's note

All claims expressed in this article are solely those of the authors and do not necessarily represent those of their affiliated organizations, or those of the publisher, the editors and the reviewers. Any product that may be evaluated in this article, or claim that may be made by its manufacturer, is not guaranteed or endorsed by the publisher.
